# Hybrid technique of virtual-assisted lung mapping and systemic indocyanine green injection for extended segmentectomy

**DOI:** 10.1186/s40792-020-01052-z

**Published:** 2020-10-27

**Authors:** Masahiro Yanagiya, Noriko Hiyama, Jun Matsumoto

**Affiliations:** Department of General Thoracic Surgery, NTT Medical Center Tokyo, 5-9-22 Higashi-Gotanda, Shinagawa-ku, Tokyo, 141-8625 Japan

**Keywords:** Thoracic surgery, Lung cancer, Segmentectomy, Bronchoscopy, Intersegmental plane, Indocyanine green, Virtual-assisted lung mapping, Video-assisted thoracic surgery

## Abstract

**Background:**

Various approaches have been used to assist and facilitate segmentectomy with favorable oncological outcomes. We describe a hybrid approach comprising virtual-assisted lung mapping (VAL-MAP), which is a preoperative bronchoscopic dye-marking technique, combined with systemic indocyanine green (ICG) injection.

**Clinical presentation:**

An asymptomatic 64-year-old man was referred to our department because of a lung nodule detected during his annual medical checkup. The chest computed-tomography image revealed a 16-mm, partly solid, ground-glass nodule in the left segment 4. Because the nodule was hardly palpable and deeply located between the left upper division segment and the left lingular segment, we performed VAL-MAP to facilitate extended left lingulectomy. Five dye markings were undertaken preoperatively. Surgery to remove the nodule was then conducted via complete three-port video-assisted thoracic surgery. The VAL-MAP markings were easily identified intraoperatively and helped locate the nodule. The intersegmental plane was identified by the ICG injection. The resection line was determined based on the intersegmental plane identified by the ICG injection and the site of the nodule suggested by the VAL-MAP markings. Following the resection line, we thoracoscopically achieved extended lingulectomy with sufficient surgical margins. The patient was discharged with no complications. The pathological diagnosis was adenocarcinoma in situ.

**Conclusion:**

The hybrid technique of VAL-MAP and systemic ICG injection can be useful for accomplishing successful extended segmentectomy.

## Background

Although thoracic surgeons are still awaiting the long-term results of ongoing prospective clinical studies, previous reports have shown favorable oncological outcomes of anatomical segmentectomy for early-stage non-small cell lung cancer, especially lesions that appear radiologically as a ground-glass nodule [1–4]. Compared with lobectomy, which has long been the gold standard treatment for lung cancer, segmentectomy offers various advantages, including preservation of lung parenchyma and respiratory function [2, 5].

Numerous techniques and approaches have been devised to assist segmentectomy [6]. Selective resected segmental inflation, systemic dye injection, endobronchial dye injection, and virtual-assisted lung mapping (VAL-MAP) are among those commonly used [7–12].

VAL-MAP is a safe, efficacious, bronchoscopic, multiple pulmonary dye-marking technique that uses virtual bronchoscopic navigation [7, 13]. It can be used to create multiple marks near the tumor that allow accurate identification of the tumor during subsequent surgery [14–16]. VAL-MAP facilitates the identification of impalpable pulmonary tumors, which makes it highly appropriate for pulmonary segmentectomy, because accurate localization of the tumor greatly assists the surgeon in achieving sufficient surgical margins [17].

Systemic indocyanine green (ICG) injection is a commonly used technique for the intraoperative identification of the intersegmental plane, which assists pulmonary segmentectomy [10, 18]. Systemic ICG injection has advantages over other methods of intersegmental plane identification, such as the lack of necessity for inflation or additional technical skills [6].

Here, we present a hybrid technique that combines VAL-MAP and systemic ICG injection to facilitate optimal segmentectomy. Using the advantages of both VAL-MAP and systemic ICG injection, we successfully performed an extended segmentectomy to treat a case of early-stage non-small cell lung cancer.

## Case presentation

**Fig. 1:**
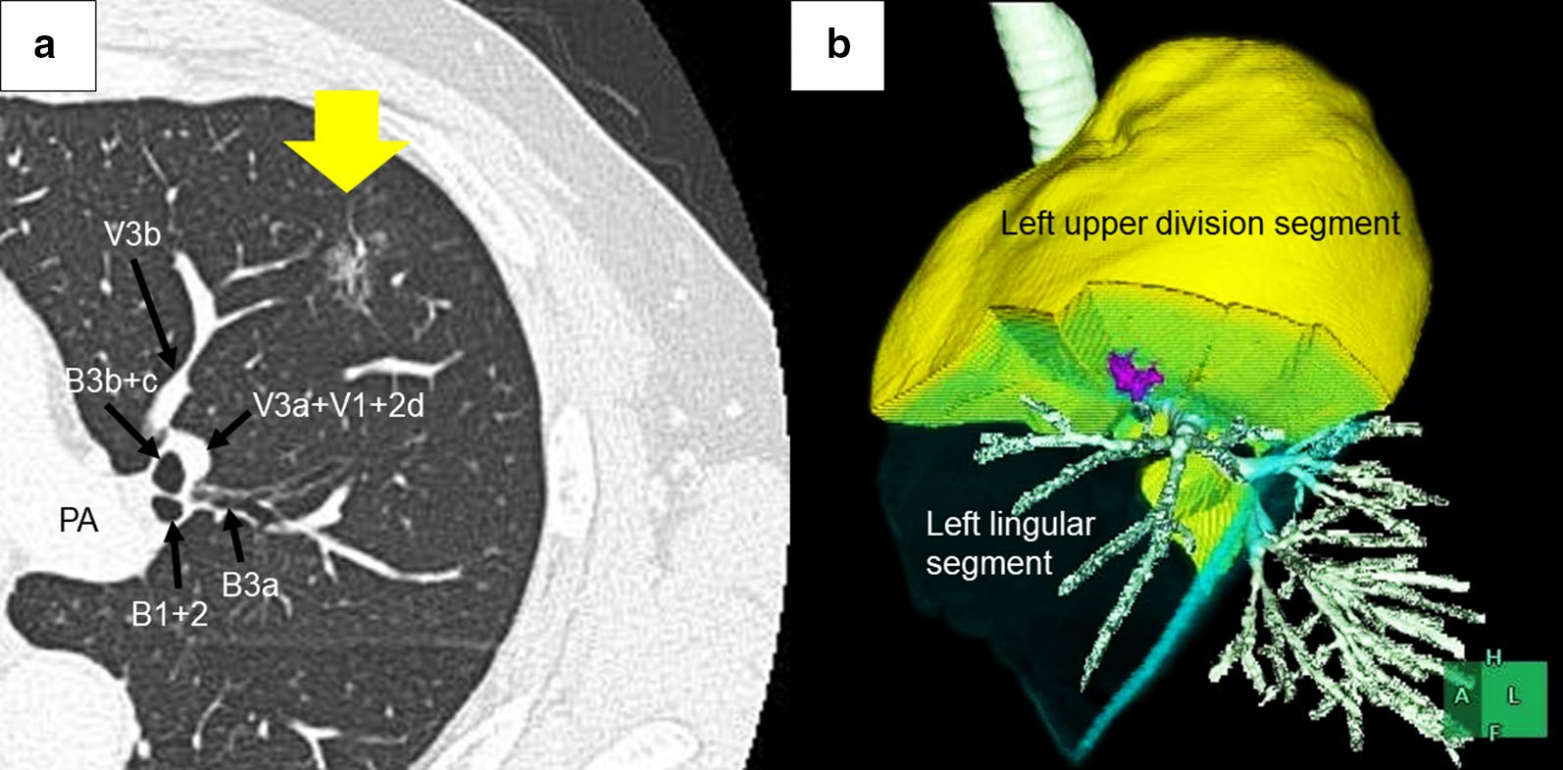
**a** Chest computed tomography reveals a partly solid, ground-glass nodule that is highly suspicious for malignancy. **b** Preoperative three-dimensional computed tomography reconstruction imaging reveals that the nodule (purple) is in the left lingular segment, but is close to the intersegmental plane between the left upper division segment and the left lingular segment

An asymptomatic 64-year-old man with no relevant medical history was referred to our hospital because of a lung nodule detected by chest computed tomography (CT) during his routine annual medical checkup. The CT image showed a 16-mm, partly solid, ground-glass nodule (the solid component measured 5 mm) in the left superior lingular segment (segment 4 (S4)) that was highly suspicious for early-stage lung adenocarcinoma (Fig. 1a). The patient was referred to our department for surgical treatment.

Although in the left S4, the nodule was near the intersegmental line between left S4 and the left upper division segments (S3 and S1 + 2) (Fig. 1b). In addition, it was deeply located (Fig. 1a). The distance between the pleura and nodule was 14 mm (Fig. 1a).

Sublobar resection was indicated because the appearance of the nodule suggested less-invasive lung cancer. There were several options for surgical procedures, including extended lingulectomy, S3 + lingulectomy, and S3 + S4 segmentectomy. Preoperative volumetric estimates were made that indicated the lingular segment to be about 523 ml, S3 + S4 to be about 717 ml, and S3 + lingular segment to be about 922 ml. To preserve the pulmonary parenchyma, we decided to perform extended lingulectomy. In addition, this patient had uncommon anatomical characteristics, such as independent B3a and B4a bronchi. Furthermore, both his upper division and lingular bronchi had three branches. S3 + S4 segmentectomy would therefore have been involved extremely complex dissection of the bronchi. For these reason, we decided to perform an extended lingulectomy to obtain a sufficient surgical margin. As the nodule was hardly palpable, preoperative marking was indicated.

On the day before the planned video-assisted thoracic surgery (VATS) to remove the nodule, we conducted VAL-MAP via bronchoscopy. Five dye markers were placed on the left lung surface as previously described [15, 16]. Under local anesthesia and mild sedation, a metal-tipped catheter (PW-6C-1; Olympus, Tokyo, Japan) was threaded through the working channel of the bronchoscope into the targeted bronchial region guided by virtual bronchoscopic navigation (Fujifilm Corporation, Tokyo, Japan). When it was confirmed by X-ray fluoroscopy that the catheter had reached the pleura, the patient was placed in a right lateral decubitus position [16]. After confirming that the tip of the catheter was in the appropriate position, we injected indigo carmine into the airways via bronchoscopy. After five such injections, chest CT images were obtained to confirm the markers’ locations and the targeted nodule. These dye markers were designed not only to pinpoint the location of the nodule, but also to suggest a resection line to help us perform the extended lingulectomy. We intended to make five marks in the peripheral branches of B3aii, B3bi, B3bii, B3c, and B4bii. Two of these marks (B3aii and B4bii) were designed to localize the tumor and two others (B3bii and B3aii) were intended to mark the intersegmental plane between the upper division and lingular segments.

**Fig. 2:**
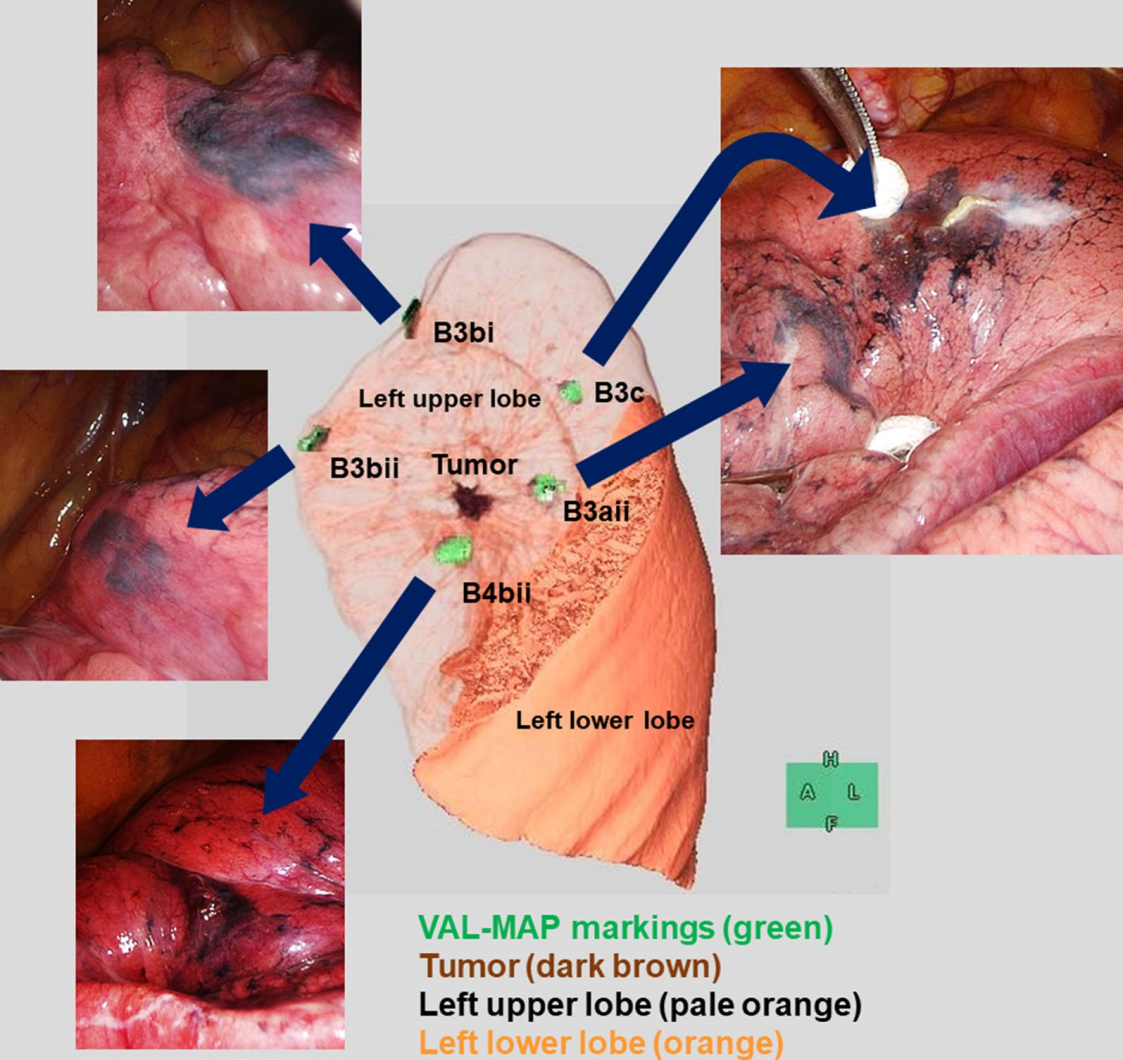
Post-mapping three-dimensional image (center) and photographs (arrows) of the mapping. Dye markings are apparent in the peripheral branches of B3aii, B3bi, B3bii, B3c, and B4bii (green). Among them, two markings (B3c and B4bii) were complicated with bulla formation. The tumor is shown in dark brown, the left upper lobe in pale orange, and the left lower lobe in orange

The surgery was conducted via a three-port VATS with the patient under general anesthesia. All VAL-MAP dye markings were highly visible. Among them, two in the peripheral branches of B3c and B4bii had accompanying bulla formation and were recorded as grade 5 according to the VAL-MAP marking grade system (Fig. 2) [15]. The other markers were recorded as grade 2 or 3 (Fig. 2) [15].

First, the interlobar fissure was assessed. Because we knew that A5 and A3a branched from the interlobar pulmonary artery, we exposed and confirmed the presence of each of these vessels. We then dissected the mediastinal pleura and confirmed the presence of branches of the pulmonary vein (PV), including V4 + 5 (lingular PV) and V3 (left upper anterior PV). Interlobar A5 was ligated and divided. Then, V4 + 5 was divided using a mechanical stapler. The lingular segmental bronchus (B4 + 5) was identified and confirmed by bronchoscopy and dissected using a mechanical stapler. Finally, A4, which branched from the mediastinal pulmonary artery, was identified and divided using the mechanical stapler.

**Fig. 3:**
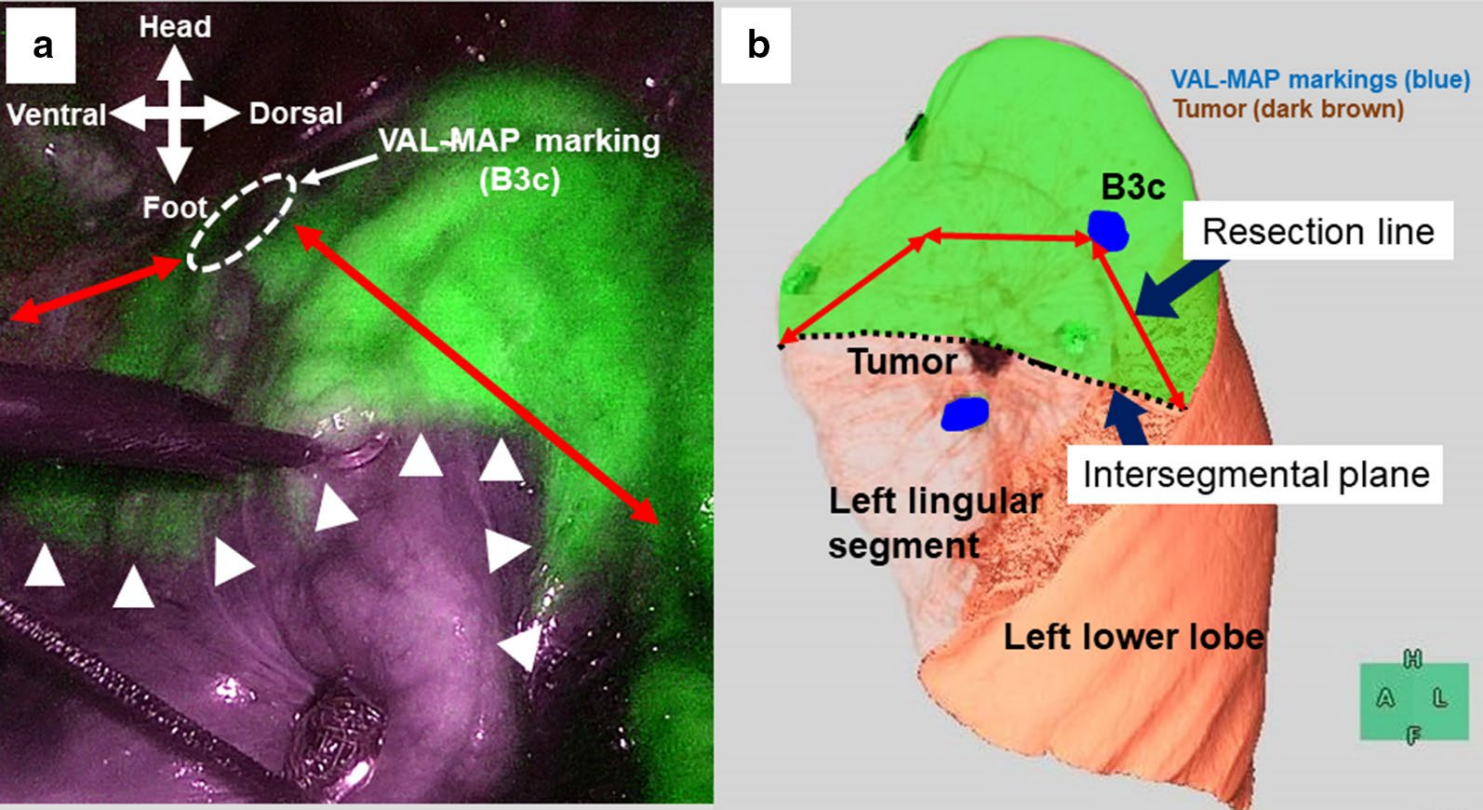
**a** Near-infrared imaging shows the delineation of the intersegmental plane (arrowheads) between the left lingular segment and the left upper division segment, created by the systemic indocyanine green injection. The white circle surrounded by dotted lines suggests a VAL-MAP mark (B3c) and the red lines show the resection lines. **b** Three-dimensional image shows the intersegmental plane and the resection line. The blue color represents the VAL-MAP marking and the dark brown area is the tumor

**Fig. 4:**
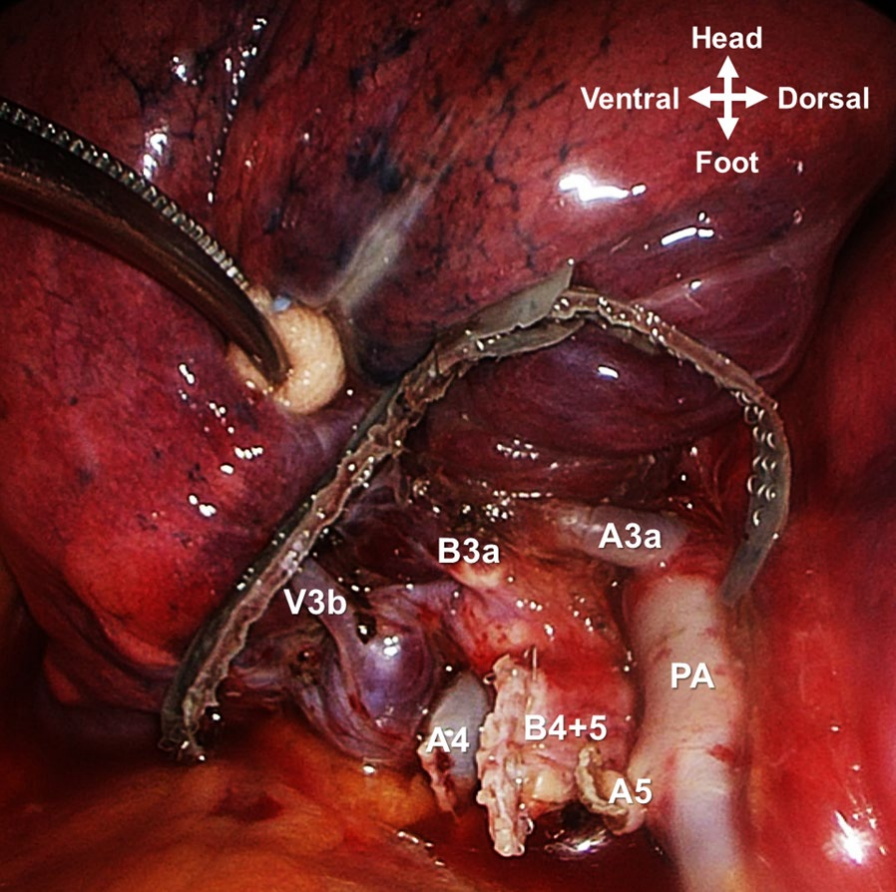
Intraoperative view of the surgical field after extended lingulectomy. *PA* pulmonary artery

The VAL-MAP markings were easily identified intraoperatively, and we were easily able to suppose the tumor localization (Fig. 2). To identify the intersegmental plane accurately, intravenous ICG was administered. The intersegmental plane was detected with the aid of near-infrared thoracoscopy (Fig. 3a). The resection line was then determined (Fig. 3b) based on the tumor location, as shown by the VAL-MAP markers, and the intersegmental plane, which became apparent after the systemic ICG injection. In mediastinal and interlobar areas, the resection line mostly followed the intersegmental plane. In the area around the tumor, the resection line was designed to maintain a certain distance from the tumor (Fig. 3b). Lung parenchyma was divided by mechanical staplers that followed the resection line. Finally, we performed the extended lingulectomy, during which not only the lingular segment was resected, but also part of the left upper division segment (Fig. 4). The surgical margin was > 2 cm. Pathological evaluation confirmed the negative surgical margin, and the pathological diagnosis was adenocarcinoma in situ. The patient was discharged on postoperative day 4 with no complications related to either VAL-MAP or the surgery. To date, the patient has survived 6 months with no recurrence and spirometry conducted 6 months after the surgery demonstrated good preservation of respiratory function (Table 1).

**Table 1:** Results of respiratory function tests before and after surgery

	Pre-operation	6 months after surgery
FVC (L)	3.86	3.74
%FVC (%)	109	106
FEV1.0 (L)	2.70	2.44
FEV1.0% (%)	69.9	65.2

## Discussion

The location of the tumor is a crucial factor for the thoracic surgeon performing a segmentectomy [17]. In the current case, the tumor was deeply located, near the intersegmental plane. If the tumor had been in a central location in the targeted segment, we would have chosen a conventional segmentectomy facilitated by systemic ICG administration. When tumors are located in a peripheral segment or near an intersegmental plane, we must consider the tumor location and segmental area with care to ensure that we can achieve a sufficient surgical margin. In the present case, we chose to perform an extended segmentectomy, which allowed concomitant resection of part of the neighboring segment [17]. Accurate knowledge of the locations of both the tumor and the intersegmental plane was necessary to achieve a successful extended segmentectomy—which is why we adopted the hybrid technique of combined VAL-MAP and systemic ICG injection.

Although VAL-MAP was initially developed to help localize tumors, it can also be used to make multiple markings near the intersegmental plane. Thus, VAL-MAP is very useful for the identification of the intersegmental plane, in addition to its use for the localization of the tumor. Hence, this technique facilitates segmentectomy. However, VAL-MAP has not always accurately identified the intersegmental plane, which has been a drawback to its use [6, 17].

Regarding identification of the intersegmental plane, systematic ICG injection has a great advantage [10, 18, 19] over VAL-MAP. Previous studies have shown that intravenous ICG injection achieved a high success rate for intersegmental plane identification [6, 19], although it does not mark tumor sites.

The hybrid technique utilizing VAL-MAP and systemic ICG injection proved efficacious in allowing the performance of an extended segmentectomy. VAL-MAP has proved competent for localizing tumors, and systemic ICG injection has proved useful for identifying the intersegmental plane. Therefore, the two methods complement one another, thereby facilitating extended segmentectomy. In addition, neither method requires lung inflation, leaving added working space for VATS [6]. This hybrid technique thus makes it easier to perform complex procedures such as extended segmentectomy, even under VATS.

Although we proved the efficacy and feasibility of the hybrid technique, there is still room for improvement. In the current case, we left five VAL-MAP markings. Because we were unsure whether the ICG injection could work, we made marks that suggested the intersegmental plane (B3bii and B3aii). Given that our hybrid technique is feasible, we may, in the future, reduce the number of VAL-MAP markings. Theoretically, such reduction might decrease the incidence of complications, such as pneumothorax or pulmonary bleeding. For future segmentectomies, we plan to leave only two or three markings near the tumor for easy localization and rely on ICG injection to establish the intersegmental plane.

This study revealed two limitations of the hybrid technique. The first is the increased time and effort needed to prepare for the surgery. VAL-MAP requires adequate preoperative preparation [6]. The second is the need for special equipment [6]. VAL-MAP requires a catheter and virtual bronchoscopic navigation, and the ICG injection requires near-infrared thoracoscopy [6, 13]. Although the hybrid technique is relatively costly, we believe that accomplishing an ideal extended segmentectomy is worth the cost of special equipment.

## Conclusions

Extended lingulectomy via complete VATS with the aid of a hybrid technique that comprises VAL-MAP and systemic ICG injection can be undertaken successfully. So far, this hybrid approach has proved useful for achieving ideal extended segmentectomy.

## Abbreviations

CT: Computed tomography
FVC: Forced vital capacity
FEV: Forced expiratory volume
ICG: Indocyanine green
PV: Pulmonary vein
VAL-MAP: Virtual-assisted lung mapping
VATS: Video-assisted thoracic surgery
